# A Case of Sacral Osteomyelitis Causing Ascending Spinal Canal Infection

**DOI:** 10.7759/cureus.67628

**Published:** 2024-08-23

**Authors:** Steven J Laxton, Dexter Woods

**Affiliations:** 1 Emergency Medicine, Ascension St Thomas Hospital, Murfreesboro, USA

**Keywords:** severe sepsis, sepsis, sacral ulcer, sacral wound, pelvic osteomyelitis, osteomyelitis treatment, osteomyelitis, sacral osteomyelitis, sacral infection, spinal infection

## Abstract

Sacral osteomyelitis is an infection of the bone that extends posterior to the pelvis at the base of the spine. This condition typically occurs in elderly or bedbound/immobile patients and is treated with wound debridement, wound care, surgery, and antibiotic therapy. In this report, a case is presented of a rare complication of sacral osteomyelitis where the infection extended through the bone into the spinal canal causing an ascending spinal canal infection. This report is meant to provide an individual example of this rare complication which can hopefully be used to improve surveillance, treatment, and outcomes of this rare condition.

## Introduction

Osteomyelitis is defined in medical literature as inflammation of the bone or bone marrow typically caused by bacterial or fungal infections [1]. Sacral osteomyelitis typically occurs following the formation of a pressure ulcer or wound overlying the sacrum or coccyx [2] which occurs in bedbound or immobile patients who do not experience frequent rolling to prevent pressure wound formation or do not have adequate mattress support. 

This case presentation has a classic story of a waxing and waning course of sacral osteomyelitis following a sacral decubitus ulcer that slowly progressed, worsened, and failed outpatient wound therapy and antibiotic therapy. Upon presentation, the patient had terminal pelvic osteomyelitis with sacral osteomyelitis that caused erosion of bone, introduced infection into the spinal canal and began to ascend the spinal canal. Surgery was considered in this case but given the poor quality of life and patient's previous expressed desire for non-escalation of therapy he was eventually transitioned to comfort care and ultimately expired in 48 hours. 

## Case presentation

A 76-year-old male presented to the emergency department following transfer from a nursing home where the patient has resided for many years for concern of fever and worsening of the chronic sacral wound. The patient is an inadequate historian secondary to dementia and nonverbal status from prior cerebrovascular accident (CVA). Additional history was gathered from the medical record and discussion with EMS personnel. The patient had a complicated past medical history with multiple comorbidities notable for advanced dementia, diabetes mellitus, peripheral arterial disease, gastroesophageal reflux disease (GERD), hypertension, CVA, right leg osteomyelitis requiring above-the-knee amputation, prior left leg above the knee amputation from peripheral arterial disease complications, gastroesophageal reflux disease, hepatitis C, depression, anxiety, post-traumatic stress disorder, and oropharyngeal dysphagia requiring placement of percutaneous endoscopic gastrostomy (PEG) tube. This is his fourth admission to our hospital this year (as of February 2023). 

Upon presentation, the patient’s vital signs were temperature 97.4F, heart rate 91 beats per minute, blood pressure 97/50 mmHg, and respiratory rate 15 with 100% oxygen saturation on room air. 

Patient had a physical examination that revealed a pleasantly demented 76-year-old male resting in bed and non-verbal but unchanged from baseline. His abdomen was significant for a PEG tube in place in epigastrium with clean skin surrounding. Patient had bilateral above-the-knee amputations (AKA) with the right AKA having surgical staples in place from recent amputation due to osteomyelitis. The patient’s skin surrounding the sacrum showed a large sacral decubitus ulcer that measured approximately 25x25 cm (about 10x10 inches) with malodorous drainage, necrotic tissue, and exposed bone, sacrum, noted at base of the wound with concern for acute worsening from baseline.

Laboratory examination showed unrevealing electrolyte panel with normal renal function and non-elevated lactic acid. Complete blood count showed a leukocytosis of 20,800 white blood cells (WBC)/mcL with neutrophilic predominance which was significantly changed from two weeks prior when his white blood cell count measured at 8500 WBC/mcL. The patient also had a depressed hemoglobin of 9.3 gm/dL that was unchanged from baseline chronic anemia. Blood cultures were drawn at initial presentation prior to administration of antibiotic therapy that produced *Proteus mirabilis* with extended-spectrum beta-lactamase producer. 

**Figure 1:**
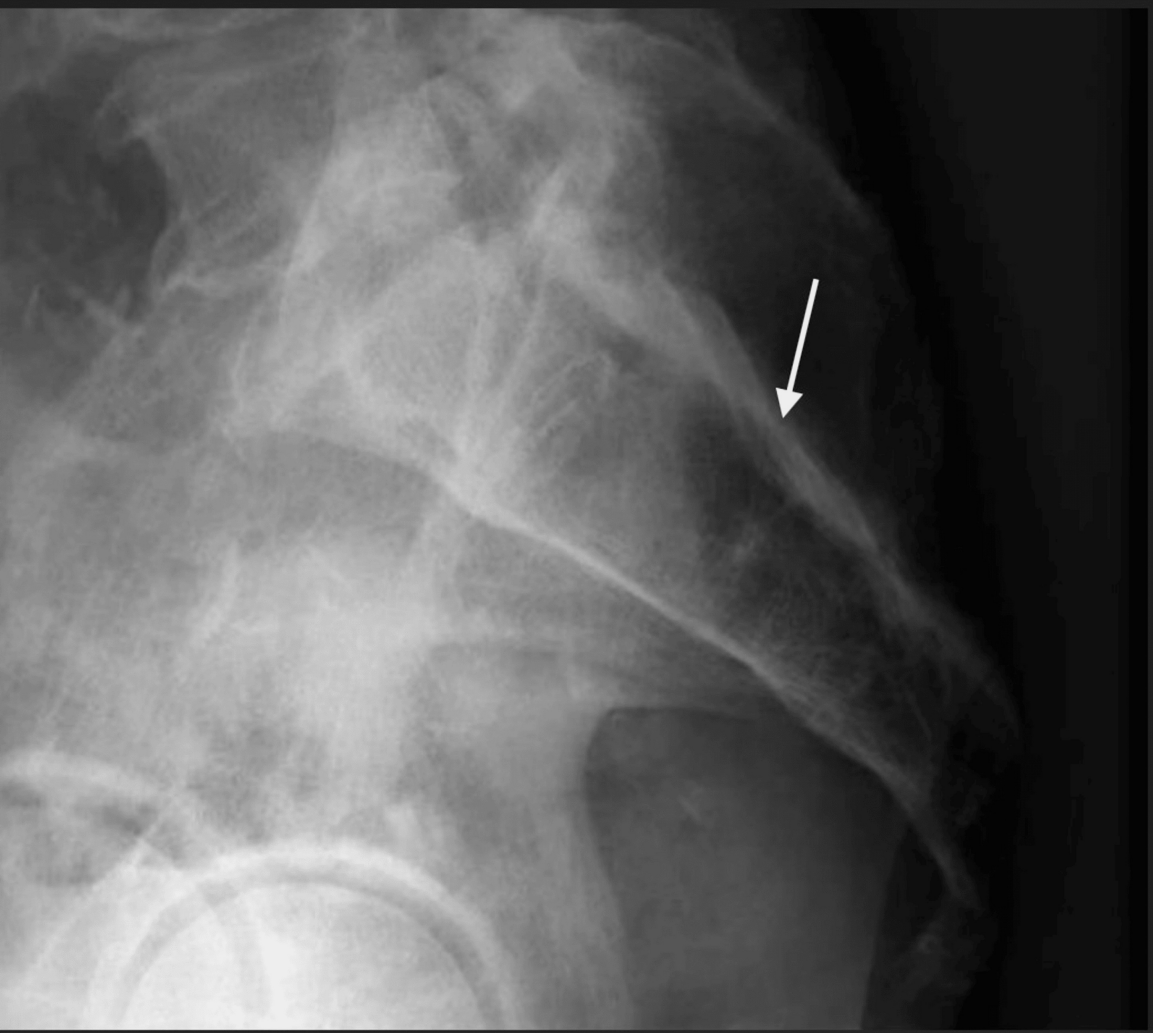
Plain film X-ray of sacrum Plain film X-ray lateral view of the sacrum showing osteomyelitis of the distal sacrum/coccyx (white arrow)

Radiologic examination was performed with plain film. Plain film radiograph showed suspicion of osteomyelitis of sacrum and coccyx however the clinical appearance suggested a more invasive infection (Figure 1) prompting advanced imaging.

**Figure 2:**
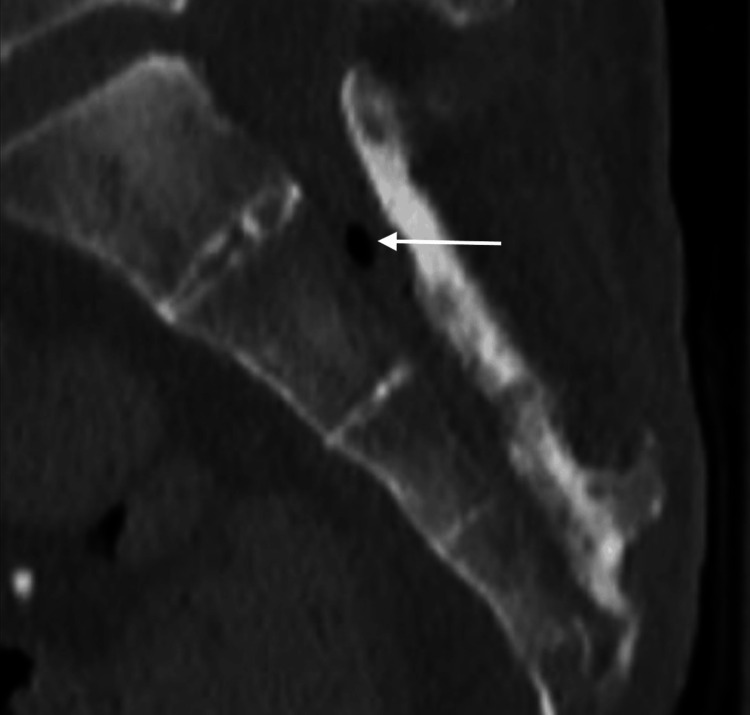
CT scan of sacrum and coccyx at additional sagittal plane Osteomyelitis of sacrum and coccyx with gas formation in spinal canal visualized on CT at different sagittal plane with evidence of ascending spinal canal infection (white arrow).

**Figure 3:**
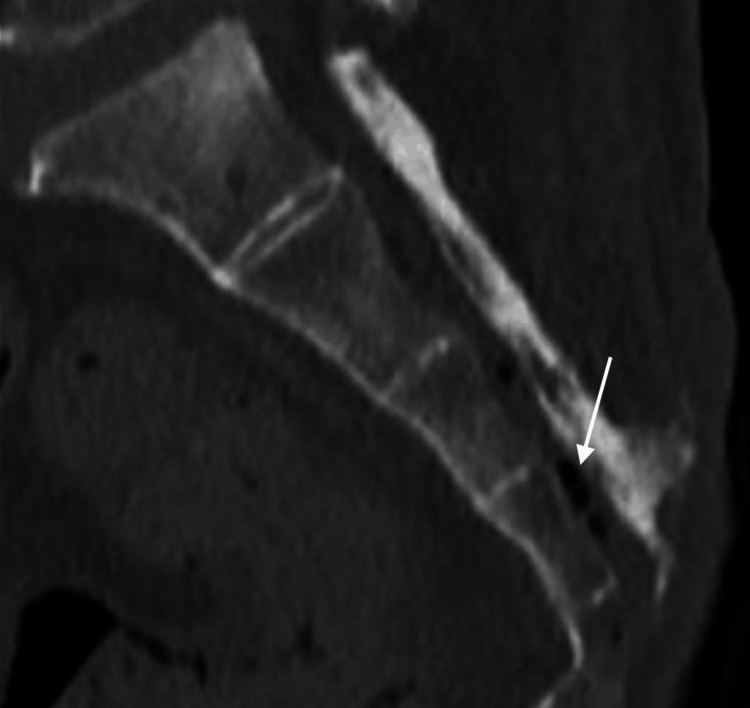
CT scan of distal sacrum and coccyx CT scan of sacrum and coccyx showing osteomyelitis and erosion with gas formation in spinal canal suggesting progressive worsening of condition with introduction of infection into spinal canal (white arrow).

**Figure 4:**
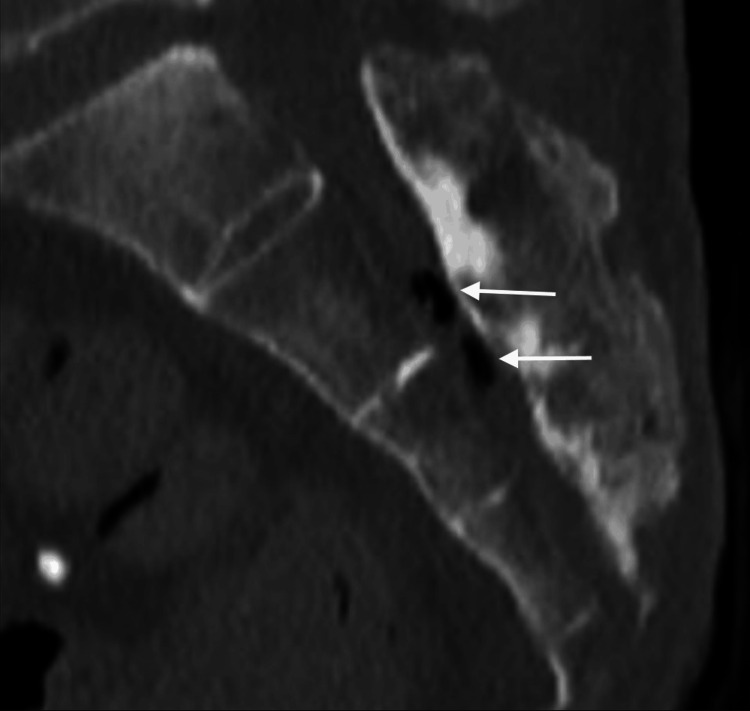
CT scan of sacrum and coccyx at different sagittal plane Osteomyelitis of distal sacrum and coccyx with gas formation in spinal canal visualized on CT at a different sagittal plane level with gas ascending the spinal canal suggesting ascending spinal canal infection (white arrows).

A computed tomography (CT) scan of the pelvis was then performed to evaluate the extent of osteomyelitis which showed extensive disease process. The CT showed destruction of the distal coccyx and distal sacrum consistent with osteomyelitis. It also showed gas formation within the sacral spinal canal. The following images are at different sagittal planes of the sacrum and coccyx showing the gas formation suggesting an ascending spinal canal infection (Figures 2-4). 

Following laboratory and radiologic examination the findings were concerning for acute on chronic worsening of sacral decubitus ulcer and osteomyelitis with interim development of ascending spinal canal infection. The patient was then started on broad-spectrum antibiotics of vancomycin and piperacillin tazobactam. Plastic surgery was consulted and recommended a goal of care discussion with the family given poor prognosis. The family then decided to transition to comfort care instead of pursuing aggressive medical therapy. 

The patient was then transitioned to comfort care and aggressive medical intervention was then discontinued. Patient passed at the nursing facility where he resided 48 hours after presentation to the emergency department. 

## Discussion

Pathology of osteomyelitis 

Osteomyelitis is defined in medical literature as inflammation of the bone or bone marrow typically caused by bacterial or fungal infections [1]. Sacral osteomyelitis typically occurs following the formation of a pressure ulcer or wound overlying the sacrum or coccyx [2] which occurs in bedbound or immobile patients that do not experience frequent rolling to prevent pressure wound formation or do not have adequate mattress support. 

**Figure 5:**
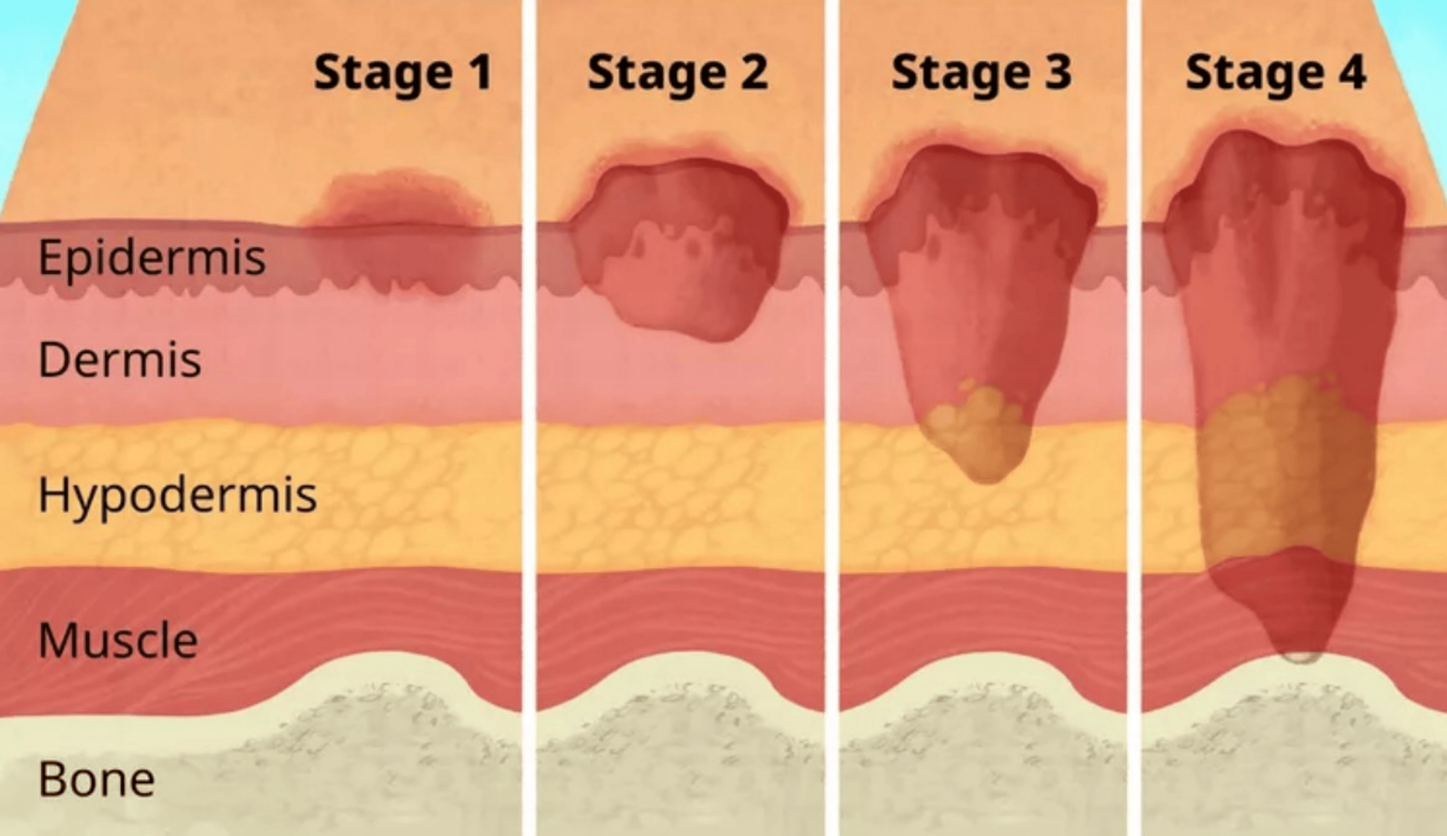
Stages of pressure ulcers Stages of pressure ulcers showing stage one through stage four with stage four being most advanced stage with bone exposure as well as a significant increase in risk of experiencing worsening infection. Image obtained with permission for republication from original author [3].

The sacral wounds that precede this condition are graded from stage one to four with stage four being the most progressed to the bone causing osteomyelitis (Figure 5).

Other predisposing factors for osteomyelitis include bacteremia due to Staphylococcus aureus, trauma (including athletic injuries, open fractures, and/or instrumentation of the pelvic bones), pressure ulcers of the sacrum, obstetrical injuries or abortion, injection drug use, urogynecologic surgery, particularly involving procedures such as bladder suspension with placement of bone anchors, other pelvic surgical procedures, including prostatectomy or transrectal prostate biopsy, cardiac catheterization, especially when performed via the inguinal/femoral site, pelvic radiation complicated by osteoradionecrosis, spinal or anal surgery, epidural anesthesia [3]. However, in sacral osteomyelitis the predominant predisposing factor is pressure ulcers. 

When osteomyelitis in the pelvis occurs, the offending microbe varies by location and cause. Hematogenous causes are commonly due to S. aureus infections [4]. Intravenous drug users that develop osteomyelitis are more likely due to pseudomonal microbes. Infections that follow pressure ulcers are mixed microbial but predominate in bowel microbes including gram negative flora [5].

Patients that present for evaluation with sacral osteomyelitis can have symptoms that vary widely. The symptoms can include vague to severe pain, febrile or afebrile, and can include poor gait, a new or worsening limp, or buttock pain. 

For definitive diagnosis culture or histopathologic examination is required and typically obtained during surgical debridement. Other nonspecific signs of osteomyelitis can include elevated white blood cell count and elevated inflammatory markers (erythrocyte sedimentation rate (ESR) and C-reactive protein (CRP)) but these are non-specific tests that can also be elevated in a myriad of other conditions. 

Further diagnostic testing can include imaging. The gold standard for the diagnosis of osteomyelitis remains in using MRI (magnetic resonance imaging), however, most clinicians start with plain film radiography, although this lacks sensitivity for diagnosing osteomyelitis. CT is more sensitive in detecting pelvic osteomyelitis but remains inferior to MRI in diagnostic accuracy [6].

Treatment

The treatment of sacral osteomyelitis often requires surgical debridement alongside antibiotic therapy and continued wound care [7]. In these cases, surgical debridement may be repeated multiple times including prolonged wound care and antibiotic therapy. The typical course of antibiotic therapy is usually from six to eight weeks, which is associated with its own risk factors. Yet, the optimal duration of antibiotic therapy remains uncertain. Antibiotic therapy is directed against staphylococcus species, gram negative bacillus, and anaerobes [8].

Despite debridement and antimicrobial therapy many ulcers of the sacrum will recur and can be associated with the need for repeat surgical wound debridement, wound care, and antibiotic therapy or intervention with advanced therapies in the cases of terminal pelvic osteomyelitis. Some of the advanced therapies can include debridement and flap creation [9] to cover wound or more invasive procedures such as hemicorporectomy which is a morbid procedure that involves the removal of the pelvis, lower extremities, bladder, rectum, and genitalia with the creation of colostomy and urostomy. This procedure is also associated with increased mortality [10].

Complications

Direct complications can include peri-wound soft tissue abscesses that can go undetected but are usually found through advanced imaging, such as CT or MRI. More severe complications, like in this case, can be contiguous spread of infection through the bone into deeper tissue spaces such as the spinal canal causing ascending spinal canal infections, spinal epidural abscesses, and/or vertebral osteomyelitis [7]. When these complications occur the mortality rate significantly increases to reported rates of around 30% within 60 days [7].

Sacral wounds are frequent complications among elderly and immobilized patients which can present from as little as a superficial skin abrasion to full erosion of skin, muscle, and ligaments with visible bone at the base of the wound. When a wound progresses to visible bone (or probe-to-bone) it is then considered osteomyelitis. 

In most cases, osteomyelitis can be treated with surgical debridement and antibiotic therapy; however, in some cases, more radical approaches are taken but are associated with increased risk [11].

In the case we presented, the patient’s course is like many others of a wound that waxes and wanes over time, resolves and relapses. The patient suffered from terminal sacral osteomyelitis in which case surgical debridement could help but would likely suffer from the need for advanced surgery such as a hemicorporectomy as the debridement could cause the dural membrane to be exposed [12,13].

## Conclusions

Sacral pressure wounds are common complications in elderly and immobilized patients that can cause significant morbidity and mortality. Infrequently, these sacral pressure wounds can progress to infection of the sacrum causing osteomyelitis which is treated with surgical debridement and prolonged antibiotic therapy and in cases that are refractory more invasive surgical procedures can be performed. In this case report, an advanced sacral wound with osteomyelitis was presented with a complication of extension into the spinal canal with gas formation and increased the disease morbidity and mortality significantly. The family decided to transition to comfort care as the patient’s illness was so far advanced, and he expired two days following hospital discharge. 

When evaluating a patient with a sacral wound that progresses to osteomyelitis of the sacrum a surgeon should be consulted for debridement and further investigation should be performed with advanced imaging, CT or MRI, to evaluate the extent of progression of osteomyelitis or non-obvious soft tissue abscess surrounding the wound. Broad-spectrum antibiotic therapy should also be employed with close wound surveillance. 
